# Perceptions Associated with Noncompliance to Community-Wide Mass Drug Administration for Soil-Transmitted Helminths

**DOI:** 10.4269/ajtmh.23-0176

**Published:** 2023-08-21

**Authors:** Kulandaipalayam Natarajan Sindhu, Kumudha Aruldas, Saravanakumar Puthupalayam Kaliappan, Elavarasan Mahendran, Judd L. Walson, Arianna Rubin Means, Sitara Swarna Rao Ajjampur

**Affiliations:** ^1^ The Wellcome Trust Research Laboratory, Division of Gastrointestinal Sciences, Christian Medical College, Vellore, India;; ^2^ Department of Global Health, University of Washington, Seattle, Washington;; ^3^ Department of Medicine, University of Washington, Seattle, Washington;; ^4^ Department of Pediatrics, University of Washington, Seattle, Washington;; ^5^ Department of Epidemiology, University of Washington, Seattle, Washington;; ^6^ The DeWorm3 Project, University of Washington, Seattle, Washington

## Abstract

Mass drug administration (MDA) is a key strategy for the control of soil-transmitted helminths (STHs). Within MDA programs, poor and non-random compliance threaten successful control of STHs. A case-control study was conducted comparing perceptions among non-compliant participants with compliant participants during a community-wide MDA (cMDA) with albendazole in southern India. Common reasons cited for non-compliance were that the individual was not infected with STH (97.4%), the perception that he/she was healthy (91%), fear of side-effects (12.8%), and dislike of consuming tablets (10.3%). Noncompliance was associated with poor awareness of intestinal worms (odds ratio [OR]: 9.63, 95% CI: 2.11–43.84), the perception that cMDA was only required for those with worms (OR: 2.14, 95% CI: 1.06–4.36), and the perception that the drug is not safe during pregnancy (OR: 2.19, 95% CI: 1.18–4.07) or when on concomitant medications (OR: 3.14, 95% CI: 1.38–7.15). Understanding of perceptions driving noncompliance can provide valuable insights to optimize participation during MDA for STHs.

In India, ongoing mass drug administration (MDA) programs for neglected tropical diseases include lymphatic filariasis (LF; currently in 256 districts in 21 states) and soil-transmitted helminths (STHs; National Deworming Day programs).^1^ A systematic review of India’s LF MDA program revealed a large coverage-compliance gap, with compliance rates ranging between 20.8% and 93.7%, with an effective compliance of ≥ 65% being reported in just 10 of the 31 rounds of MDA.^2^ Both programmatic as well as individual-level factors have been shown to influence noncompliance to MDA.^3^ Fear of side effects and fear of adverse reactions were the predominant reasons associated with noncompliance to albendazole/diethylcarbamazine in LF programs implemented across various regions of India. Other factors included a lack of faith in or motivation to take the drug, concurrent illnesses, and misinformation conveyed because of inadequate community sensitization activities.^4^^–^^8^ A study from Nepal showed that compliance during LF MDA was associated with awareness of the treatment drug, side effects, and MDA campaigns and visits by healthcare workers.^9^ Another study in Tamil Nadu state in southern India reported significantly increased coverage and compliance in the LF program when a strict protocol that mandated three home visits by drug distributors was implemented.^10^

For STHs, targeted deworming of at-risk populations is the current WHO-recommended strategy for control of STHs and associated morbidities. Mathematical models suggest that high coverage community-wide MDA (cMDA), irrespective of age, gender, or socioeconomic status, may lead to interruption of STH transmission.^11^ The DeWorm3 study is a large cluster-randomized trial being conducted in three countries (Benin, India, and Malawi) to test the feasibility of cMDA versus school-based targeted deworming (albendazole delivered biannually) for interrupting transmission of STHs.^12^ To understand the perceptions associated with noncompliance to cMDA in the India DeWorm3 site, this case-control study was conducted during the second year of the trial, after the third of a total of six rounds of cMDA.

In India, the Deworm3 trial site is located in two blocks in the southern state of India in Tamil Nadu, namely Timiri, a rural block in the Ranipet district (formerly Vellore district), and Jawadhu Hills, a tribal block in the Tiruvannamalai district. The trial site was demarcated into 20 intervention and 20 control clusters, and the characteristics of the study population and prevalence of STHs in this community have been described earlier.^13^ The cMDA was directly observed treatment delivered house-to-house and during each round of cMDA; compliance and noncompliance to treatment of each individual who was eligible and available for treatment in the enumerated population were captured. A matched case-control study design (case: control = 1:1) with a sample size of 100 cases and 100 controls (imputing an expected odds ratio [OR] = 2.5, α = 0.05, β = 80%, 51% exposure) was used for conducting this survey on noncompliance (Figure 1). Noncompliant individuals (cases) and compliant individuals (controls) aged ≥ 18 years during cMDA round 3 (cMDA3) (who may or may not have been compliant in the previous two rounds of cMDA) and were available during all three cMDA rounds were included in the survey. Controls, matched for age, sex, and village of residence from the baseline census data conducted between December 2017 and February 2018 were selected by random sampling.

**Figure 1.**
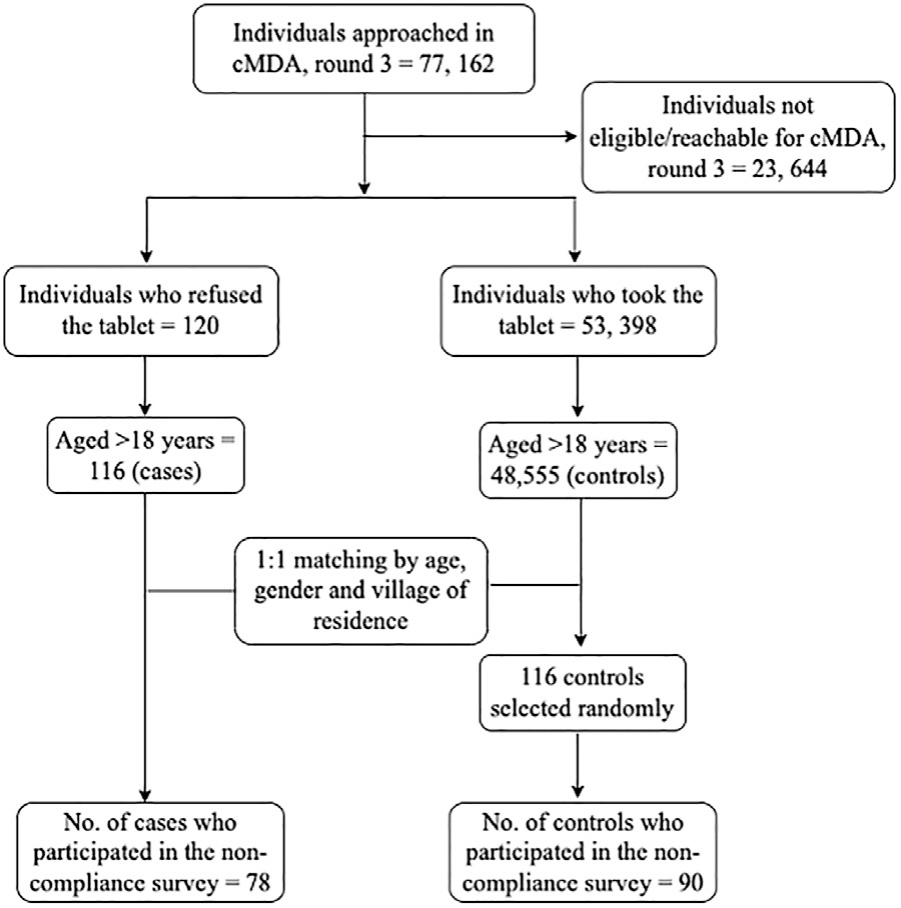
Flowchart depicting the selection of cases and controls from the community-wide mass drug administration (cMDA), round 3, for the noncompliance survey.

A pilot-tested, semi-structured questionnaire was administered by three trained field research assistants in the local language (Tamil), and data were collected using an android-based mobile platform used for all Deworm3 surveys (SurveyCTO; Dobility, Inc., Cambridge, MA, and Ahmedabad, India).^14^ Fisher’s exact or χ^2^ tests were performed as appropriate to determine the association between noncompliance, and exposure variables and ORs with 95% CI were calculated from univariable logistic regression analyses.

The Deworm3 trial was approved by the Institutional Review Board (IRB) of Christian Medical College, Vellore (CMC) [IRB Min No: 10,392] and the Human Subjects Division at the University of Washington (STUDY00000180). This analysis was approved as an amendment at CMC (A07-27.03.2019). The trial is registered at ClinicalTrials.gov (NCT03014167). Written informed consent was obtained from the individuals participating in this survey.

During cMDA3 in February 2019, a total of 53,518 individuals out of 77,162 individuals in the intervention clusters were eligible or available to participate. Of these 77,162 individuals, 23,644 individuals could not be treated either because they were not eligible or available, and 120 individuals were documented as noncompliant to treatment (0.2%) (Figure 1). Most refusals to MDA (noncompliant) were in the Timiri block, with very few refusals documented in the Jawadhu Hills block (4/120). Of the 116 adult noncompliant individuals during cMDA3, 78 individuals (cases) spread across 15 intervention clusters consented to participate in this survey along with 90 matched compliant individuals (controls) (Figure 1). The median age of the participants in both groups was similar (case: median age = 44 years, interquartile range = 28.8–61.2 years and control: median age = 46 years, interquartile range = 29.1–59 years). The proportion of males and females among the cases and controls was also comparable (Table 1). More than 80% of the interviews were administered at the participants’ place of residence, and this was documented to determine whether the interviewees, specifically the noncompliant individuals, were comfortable in their home environment and were able to respond without any external influence during the interview. About 37% of the noncompliant individuals belonged to a higher socioeconomic quintile (> 5), whereas 44% of the compliant individuals belonged to lower socioeconomic quintiles (3 and 4). A majority of both noncompliant (94%) and compliant (92%) individuals were aware that the deworming tablet was distributed to everyone in the community during the DeWorm3 cMDA to ensure individuals are healthy or to eliminate worms.

**Table 1:** Baseline characteristics of participants who took part in the noncompliance survey (*N* = 168)

Characteristic		Cases (noncompliant, *N *= 78) *n *(%)	Controls (compliant, *N *= 90) *n *(%)	*P* value
Age group	18–24 years	13 (16.7)	14 (15.6)	0.97
	25–34 years	20 (25.6)	22 (24.4)	
	35–44 years	8 (10.3)	8 (8.9)	
	> 45 years	37 (47.4)	46 (51.1)	
Gender	Male	38 (48.7)	44 (48.9)	0.98
	Female	40 (51.3)	46 (51.1)	
Socioeconomic quintile*†	1–2	21 (28)	14 (15.9)	0.15
	3–4	26 (34.7)	39 (44.3)	
	> 5	28 (37.3)	35 (39.8)	

* Quintiles, where 1 = Low and 5 = High (the wealth index was assessed using quintiles derived from household assets using principal component analysis as described elsewhere.^13^

† Data not available for three cases and two controls.

The reasons for noncompliance included 1) feeling that they were not infected with STHs (97.4%); 2) being apparently healthy (91%); 3) fear of side effects due to the albendazole tablet distributed during the cMDA (12.8%) or because of underlying health conditions or being on concomitant medications (15.4%); 4) dislike of consuming tablets (10.3%); and 5) pregnancy or breastfeeding at the time of cMDA (7.7%) (Supplemental Table 1). About one-third of noncompliant individuals (37%) and a quarter of compliant individuals (25%) were on regular medication at the time of the survey. A higher proportion of noncompliant individuals (16%) had a history of an unpleasant experience after consuming any medication compared with compliant individuals (11%).

Factors significantly associated with noncompliance were 1) not having consumed deworming medications in the past (OR: 8.94, 95% CI: 1.95–40); 2) being unaware of what intestinal worms are (OR: 9.63, 95% CI: 2.11–43.84); and 3) a perception that it was unsafe to consume deworming tablets during a range of conditions such as 1) while not having worms (OR: 2.14, 95% CI: 1.06–4.36); 2) during pregnancy (OR: 2.19, 95% CI: 1.18–4.07); 3) while on concomitant medications (OR: 3.14, 95% CI: 1.38–7.15); 4) having skin allergies (OR: 2.46, 95% CI: 1.31–4.60); 5) consuming traditional medicines (OR: 2, 95% CI: 1.06–3.79); 6) having chronic conditions such as heart disease, diabetes, or hypertension (OR: 3.97, 95% CI: 2.07–7.63); and 7) having undergone recent surgery (OR: 2.14, 95% CI: 1.16–3.97) (Table 2). With reference to questions that were administered specifically to the women who participated in the survey (*N* = 86; cases = 40 and control = 46), 75% of noncompliant women and 61% of compliant women either did not know that it was safe or believed that it was unsafe for women to consume deworming tablets during menstruation (not significant). Some of them also believed that vaginal discharge could increase by consuming deworming tablets (25% of noncompliant women and 33% of compliant women; not significant).

**Table 2:** Awareness and perceptions about STHs and cMDA among compliant and noncompliant participants

Question	Response	Cases (noncompliant, *N *= 78), *n *(%)	Controls (compliant, *N *= 90), *n *(%)	Logistic regression	
				OR (95% CI)	*P* value
Have you ever taken any tablets in the past?	No	13 (16.7)	2 (2.2)	**8.94 (1.95–40)**	**0.005**
	Yes	65 (83.3)	88 (97.8)		
If yes, did you take/do you take the tablets that you mentioned regularly?	No	41 (63.1)	66 (75)	0.57 (0.28**–**1.14)	0.114
	Yes	24 (36.9)	22 (25)		
Have you had any unpleasant experience/s with the tablets you mentioned in the past?	No	55 (84.6)	78 (88.6)	0.71 (0.27-1.81)	0.477
	Yes	10 (15.4)	10 (11.4)		
Do you know what intestinal worms are?	No/does not know	14 (17.9)	2 (2.2)	**9.63 (2.11–43.84)**	**0.003**
	Yes	64 (82.1)	88 (97.8)		
Why do you think a deworming tablet was given to everyone in the village?	For all to be healthy/eliminate worms	72 (92.3)	85 (94.4)	1.41 (0.42**–**4.84)	0.578
	Other/does not know	6 (7.7)	5 (5.6)		
Do you think a person who does not have worms can still take the deworming tablet?	No/does not know	26 (33.3)	17 (18.9)	**2.14 (1.06–4.36)**	**0.034**
	Yes	52 (66.7)	73 (81.1)		
Do you think the deworming tablet distributed to everyone has any side effects?	No/does not know	65 (83.3)	82 (91.1)	2.05 (0.80**–**5.24)	0.134
	Yes	13 (16.7)	8 (8.9)		
What do you think about the quality of the deworming tablets?	Bad quality/does not know	15 (19.2)	9 (10)	2.14 (0.88**–**5.21)	0.093
	Good quality	63 (80.8)	81 (90)		
Is it all right for a pregnant woman to take the deworming tablet?	No/does not know	48 (61.5)	38 (42.2)	**2.19 (1.18–4.07)**	**0.013**
	Yes	30 (38.5)	52 (57.8)		
Is it all right for a woman to take the deworming tablet while breastfeeding?	No/does not know	32 (41)	26 (28.9)	1.71 (0.90–3.25)	0.100
	Yes	46 (59)	64 (71.1)		
Is it all right for someone taking any other medicine/s to take the deworming tablet?	No/does not know	22 (28.2)	10 (11.1)	**3.14 (1.38–7.15)**	**0.006**
	Yes	56 (71.8)	80 (88.9)		
Is it all right for someone with a skin allergy to take the deworming tablet?	No/does not know	43 (55.1)	30 (33.3)	**2.46 (1.31–4.60)**	**0.005**
	Yes	35 (44.9)	60 (66.7)		
Is it all right for someone allergic to allopathic (English) medicines to take the deworming tablet?	No/does not know	47 (60.3)	43 (47.8)	1.66 (0.90–3.06)	0.107
	Yes	31 (39.7)	47 (52.2)		
Is it all right for someone taking traditional medicines (homeopathy, siddha, others) to take the deworming tablet?	No/does not know	55 (70.5)	49 (54.4)	**2 (1.06–3.79)**	**0.034**
	Yes	23 (29.5)	41 (45.6)		
Can someone with heart disease/diabetes/hypertension, etc., take the deworming tablet?	No/does not know	45 (57.7)	23 (25.6)	**3.97 (2.07–7.63)**	**< 0.001**
	Yes	33 (42.3)	67 (74.4)		
Is it all right for someone who recently underwent surgery to take the deworming tablet?	No/does not know	45 (57.7)	35 (38.9)	**2.14 (1.16–3.97)**	**0.016**
	Yes	33 (42.3)	55 (61.1)		
Is it all right for someone who is generally feeling unwell to take the deworming tablet?	No/does not know	32 (41)	26 (28.9)	1.71 (0.90–3.25)	0.100
	Yes	46 (59)	64 (71.1)		
Is it all right to take the deworming tablet on an empty stomach?	No/does not know	55 (70.5)	65 (72.2)	0.92 (0.47–1.80)	0.807
	Yes	23 (29.5)	25 (27.8)		
Is it all right for old people to take the deworming tablet?	No/does not know	21 (26.9)	16 (17.8)	1.70 (0.82–3.56)	0.156
	Yes	57 (73.1)	74 (52.2)		
Is it all right to take the deworming tablet distributed in the village without a doctor’s advice?	No/does not know	45 (57.7)	40 (44.4)	1.70 (0.92–3.14)	0.088
	Yes	33 (42.3)	50 (55.6)		
Is it all right for someone under the influence of alcohol to take this deworming tablet?	No/does not know	75 (96.2)	83 (92.2)	2.11 (0.55–8.45)	0.342
	Yes	3 (3.8)	7 (7.8)		
Questions asked only of women (cases, *N *= 40; controls, *N *= 46)					
Is it all right for a woman to take this deworming tablet when she is menstruating?	No/does not know/do not want to answer	30 (75)	28 (60.9)	1.93 (0.76–4.88)	0.166
	Yes	10 (25)	18 (39.1)		
Do you think white discharge (vaginal) will increase if a woman takes this deworming tablet	No/I don’t know	30 (75)	31 (67.4)	1.45 (0.56–3.73)	0.439
	Yes	10 (25)	15 (32.6)		

cMDA = community-wide mass drug administration; OR = odds ratio; STHs = soil-transmitted helminths.

Bold values represent significant values at *P* < 0.05.

Our study had a few limitations. The survey adopted a careful approach to exclude open-ended questions to ensure participation of noncompliant individuals, but this may have limited the potential to elicit more in-depth information around noncompliance. Some of the general questions on awareness of STHs and prior deworming that are nonspecific to perceptions associated with noncompliance had wide CIs that could be attributed to the small sample size of this study. Interactions of participants with deworming providers may also be a factor influencing compliance that was not explored in this study. However, government stakeholder perspectives on MDA collected as part of the parent trial (Deworm3) indicated that availability of high-quality, tailored sensitization materials and human and material resources would be essential for implementing cMDA.^15^

In the DeWorm3 trial, the strategy of house-to-house cMDA delivery provided an opportunity for interpersonal communication with noncompliant individuals (0.2% of the study population) spread across almost three-quarters of the study clusters. This survey delineates perceptions among those noncompliant with cMDA for STHs in this community and provides insights that could help address challenges in the larger national cMDA programs for STHs in the Indian context. Previous studies from India have focused on noncompliance in LF MDA programs with little to no information on STH programs.^2^^–^^6^ The perceptions that healthy individuals should not participate in cMDA and that the drug distributed in cMDA was not safe to take in conditions such as pregnancy or in those with concomitant conditions such as heart disease, hypertension, or diabetes were the most important factors associated with noncompliance in the cMDA. Similar findings have also been shown in other studies.^16^^,^^17^ In this survey, > 90% of noncompliant individuals felt there was no need to take albendazole as they had no worms and were apparently healthy. Similar to observations during the LF MDA programs in India, fear of side effects from albendazole, either directly or associated with underlying health conditions or concomitant medications, was a common reason for treatment refusal in this survey.^18^^,^^19^ Findings from Egypt and Sri Lanka showed that reasons for noncompliance to MDA included not feeling the need for treatment, the fear of adverse effects, pregnancy or breastfeeding, being on concomitant medications, or having forgotten to take the drug.^16^^,^^20^ Treatment compliance was associated with the perception that deworming is safe. Overall, messages stressing the safety of deworming should be included in community sensitization messages to help improve compliance to STH cMDA in similar settings in India and other countries.

## Supplemental Materials


Supplemental materials
