# Supplementary material for: Perceptions Associated with Noncompliance to Community-Wide Mass Drug Administration for Soil-Transmitted Helminths
Source: Am J Trop Med Hyg. 2023 Aug 21;109(4):830–4. doi: 10.4269/ajtmh.23-0176 (PMC10551087; doi:10.4269/ajtmh.23-0176)
Supplement: Supplementary file 1 [file tpmd230176.SD1.pdf]

Supplementary Table 1

Reasons for non-compliance during one or more rounds of community-wide mass drug administration*

**Variable**	***N=78***<br>***n (%)***
I have no worms, so why the need for this tablet	76 (97.4)
I am healthy now, so why the need for this tablet	71 (91)
I might have side-effects after taking this tablet	10 (12.8)
I am taking other medicines	10 (12.8)
I don’t like to swallow tablets	8 (10.3)
I might have diarrhoea after taking this tablet	4 (5.1)
I currently have a skin allergy	4 (5.1)
I am allergic to allopathic medicines	4 (5.1)
I believe in traditional medicines only	4 (5.1)
I have undergone a surgery in the past	4 (5.1)
I am too old for such medications	4 (5.1)
I was pregnant at the time of this tablet distribution	4 (5.1)
I was breastfeeding at the time of this tablet distribution	2 (2.6)
I was unwell at the time of this tablet distribution	2 (2.6)
I have a chronic illness (heart disease/diabetes/hypertension)	2 (2.6)
I was shy to take the tablet in front of the health worker when offered	1 (1.3)

*Multiple reasons can be selected as a response by the subject
